# A novel performance monitoring framework for health research systems: experiences of the National Institute for Health Research in England

**DOI:** 10.1186/1478-4505-9-13

**Published:** 2011-03-24

**Authors:** Anas El Turabi, Michael Hallsworth, Tom Ling, Jonathan Grant

**Affiliations:** 1 Cambridge Centre for Health Services Research, Institute of Public Health, University of Cambridge, Robinson Way, Cambridge, CB2 0SR, UK; 2 AND Europe, Westbrook Centre, Milton Road, Cambridge, CB4 1YG, UK; 3 Institute for Government, 2 Carlton Gardens, London, SW1Y 5AA, UK

## Abstract

**Background:**

The National Institute for Health Research (NIHR) was established in 2006 with the aim of creating an applied health research system embedded within the English National Health Service (NHS). NIHR sought to implement an approach for monitoring its performance that effectively linked early indicators of performance with longer-term research impacts. We attempted to develop and apply a conceptual framework for defining appropriate key performance indicators for NIHR.

**Method:**

Following a review of relevant literature, a conceptual framework for defining performance indicators for NIHR was developed, based on a hybridisation of the logic model and balanced scorecard approaches. This framework was validated through interviews with key NIHR stakeholders and a pilot in one division of NIHR, before being refined and applied more widely. Indicators were then selected and aggregated to create a basket of indicators aligned to NIHR's strategic goals, which could be reported to NIHR's leadership team on a quarterly basis via an oversight dashboard.

**Results:**

Senior health research system managers and practitioners endorsed the conceptual framework developed and reported satisfaction with the breadth and balance of indicators selected for reporting.

**Conclusions:**

The use of the hybrid conceptual framework provides a pragmatic approach to defining performance indicators that are aligned to the strategic aims of a health research system. The particular strength of this framework is its capacity to provide an empirical link, over time, between upstream activities of a health research system and its long-term strategic objectives.

## Background

### The evolution of national health research systems

Over the last twenty years there has been a greater than five-fold increase in spending on health research globally, from an estimated US$30bn in 1986 [1] to over US$160bn in 2005 [2]. A substantial proportion of this funding has come from public sector sources (49% in 2005) [2], that is to say from governments and publicly funded charities. Along with this increased expenditure, there has been a shift in the thinking of governments regarding the role of Government in supporting health research. First, the concept of 'essential national health research' (ENHR) has gained increasing acceptance in health research policy circles following its proposal in 1990 by the Commission on Health Research for Development [3]. ENHR was originally proposed as a means of defining a research agenda focused on the priority health needs of a country, thereby explicitly tying the actions of a nation's health research resources to the goals of its health system. This coupling of national health research priorities to national health systems subsequently led to the explicit recognition of the existence and importance of 'national health research systems', a term marshalled most prominently by the WHO International Workshop on National Health Research Systems, held in Thailand in 2001. This workshop defined a national health research system as "a system for planning, coordinating, monitoring and managing health research resources and activities; and for promoting research for effective and equitable national health development" [4]. Implicit in this definition is the assumption that improved integration of health research activity into a cohesively managed national system will improve the performance of that system in meeting national health research priorities.

### Performance monitoring and evaluation

The distinction between performance monitoring and evaluation is an important one. Performance monitoring can be defined as "a continuous process of collecting and analyzing data to compare how well a project, program, or policy is being implemented against expected results" (OECD 2002) [5]. The central activity of performance monitoring is the collation of data to facilitate the reporting of performance indicators, that is to say quantitative or qualitative variables that allow for the identification of changes produced by a specific intervention, activity, project, programme or policy (OECD 2002) [5]. Performance monitoring is an integral component of performance management, whereby management control systems respond to performance information in a manner conducive to the improvement of performance [6]. Evaluation, on the other hand, can be defined as the "*judgement of interventions according to their results, impacts and needs they aim to satisfy" *(OECD 2002) [5]. In contrast to performance monitoring, evaluation (particularly in a public sector context) is often understood to be concerned with summative assessments of the success or failure of specific programmes and projects in achieving their stated goals.

Effective performance management requires managers to have access to performance information that gives a balanced view of organisational performance without overwhelming managers with data or overburdening their organisation with reporting requirements. The aim of producing a small but balanced set of indicators to support strategic decision-making has given rise in recent years to the idea of "dashboards" [7]. The purpose of a dashboard is to present a small set of performance measures on a regular and structured basis to strategic decision-makers. These performance indicators should focus attention on activities of greatest importance to an organisation and its stakeholders, thereby minimising the data collection burden.

The construction of a dashboard requires a full understanding of the operation of the organisation and the collaboration of its employees. A dashboard requires the creation of an optimal list of metrics from a large pool of potential indicators in a way that makes sense to those delivering services, as well as to strategic management and wider stakeholders. Put formally, the information architecture of a good dashboard will optimise the efficiency of an organisation's performance monitoring systems.

### Performance monitoring and evaluation of national health research systems

As governments have grasped the concept of national health research systems, some have attempted to take more strategic approaches to manage these systems for national benefit. Increased investment in research for health has been accompanied by increased interest in how this money is spent and what it achieves [8].

Governments such as those in Canada [9], Singapore [10] and Australia [11] have sought to consolidate previously disparate elements of their health research activities into cohesively managed systems, often with unified funding streams and lines of accountability. Whilst there have been attempts to develop conceptual frameworks for evaluating the functioning of national health research systems [12] and the economic contribution of research and development [13], none of these has addressed how to monitor prospectively the performance of health research systems. There have also been attempts to produce 'snapshot' assessments of the capacities and capabilities of various national health research systems [14,15]. These analyses were not, however, designed to be part of a continuous process of data collation and analysis that might inform management of the systems.

Thus, the combination of increased public funding for health research, growing political attention on national health research systems, and a paucity of empirically tested frameworks for performance monitoring of investments in such systems has created a challenge for governments seeking to strategically strengthen health research systems for national health, social and economic benefit.

### The National Institute for Health Research

In January 2006 the Government of the United Kingdom launched its health research strategy, *Best Research for Best Health *[16]. The National Institute for Health Research (NIHR) was established in April 2006 under the management of the Department of Health as the main mechanism for delivering the vision, mission and goals of the strategy. Working in concert with other health research funders, (particularly the UK Medical Research Council and members of the UK Clinical Research Collaboration), NIHR adopted a central role in creating and optimising an applied health research system embedded within the English National Health Service (NHS). Through a diverse investment programme NIHR sought to create a critical link between the health and health research systems in England.

In June 2006, NIHR published its plans for delivering a broad programme of initiatives designed to achieve the objectives of *Best Research for Best Health*. NIHR initially published sixteen implementation plans grouped under six headings (Appendix 1). Each implementation plan described a specific programme of activity designed to deliver a specific objective of *Best Research for Best Health*. Examples included plans to establish biomedical research units in health-research areas of unmet need and plans to build research workforce capacity through the creation of new training programmes.

Explicit within *Best Research for Best Health *was a commitment by the UK Government to "*implement methods of evaluating the equality and effectiveness of ... capacity, infrastructure and direct funding*". The strategy also stated that "*all elements of the new strategy will operate under clear and robust management arrangements supported by programmes of evaluation*". These commitments, taken together with pre-existing UK Government policy on performance management of public sector delivery [17] and health research in particular [18], set a clear requirement for NIHR to develop and implement a cogent approach for monitoring and evaluating its activities and performance.

Our work aimed to develop and implement a framework for defining key performance indicators that would allow NIHR to meet its reporting requirements and build its monitoring and evaluation capability.

## Methods

Between February 2007 and June 2009 we developed and piloted a framework for defining performance indicators for the National Institute for Health Research in England. To be acceptable to NIHR, our framework needed to address the inherent challenges of measuring performance in health research, namely: the long lead time between investment activity (inputs) and return on investment (outputs and outcomes) associated with health research; the non-linearity of the research process; and the effect that these factors have on attributing outcomes [19-21]. The framework also needed to allow NIHR to discharge its reporting duties in line with the commitments made in the UK Government health research strategy (Appendix 2), and we wanted to ensure compliance with FABRIC [17], a best practice framework for performance information systems developed by the UK Government.

### Development of conceptual framework

A review of relevant peer-reviewed and grey literature revealed no performance monitoring frameworks that met the specifications set. As a result, we opted to create a novel conceptual framework by combining elements of two existing performance frameworks: the logical frame (or log-frame) approach (LFA) and the Balanced Scorecard (BSC).

From the log-frame approach we adopted the logic model, simplified to inputs, processes, outputs and outcomes. This enabled us to address the challenge of tracking the longitudinal relationship between investment, system activities and system outcomes over longer time-periods. From the BSC we adopted the three domains of performance monitoring that were relevant to NIHR (financial performance, internal processes and interactions with external parties), which enabled us to take account of the non-linear interdependencies of the research process.

The 'learning and growth' domain proposed in Kaplan and Norton's original balanced scorecard was not formally incorporated into our framework for two reasons. First, we developed our framework when NIHR was in its initial growth phase, during which time it was launching and establishing multiple new initiatives. The progress of this foundation process was already being monitored through the publication and regular updating of NIHR's implementation plans. In the presence of this milestone reporting, it was felt that any new indicators would be likely to simply restate already reported levels of performance in this domain. Second, the research workforce capacity development activities of NIHR (an important area of activity that might typically be represented in the learning and growth domain) were already represented in a specific implementation plan. As a result, we judged that performance in this area would be sufficiently captured through the development of appropriate financial, internal and external performance indicators, as proposed in our framework.

**Figure 1:**
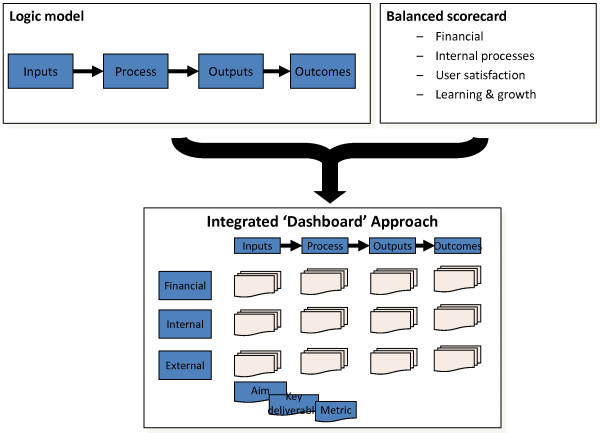
Integrated dashboard structure

By combining the logic model structure with the three BSC domains we were able to produce a structure for defining a dashboard of performance indicators (Figure 1) with the potential to improve attribution of health research system outcomes.

**Figure 2:**
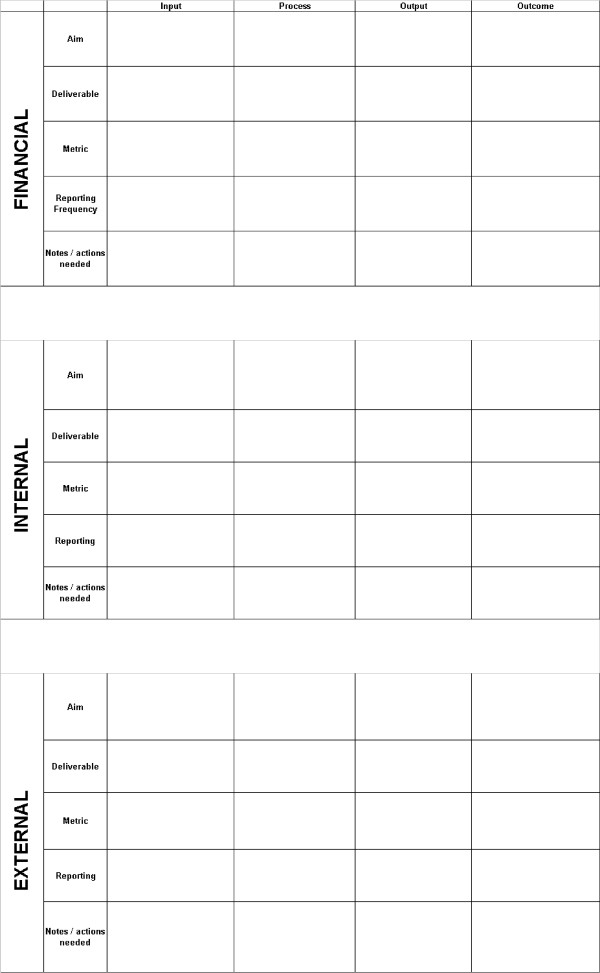
NIHR dashboard framework template

**Figure 3:**
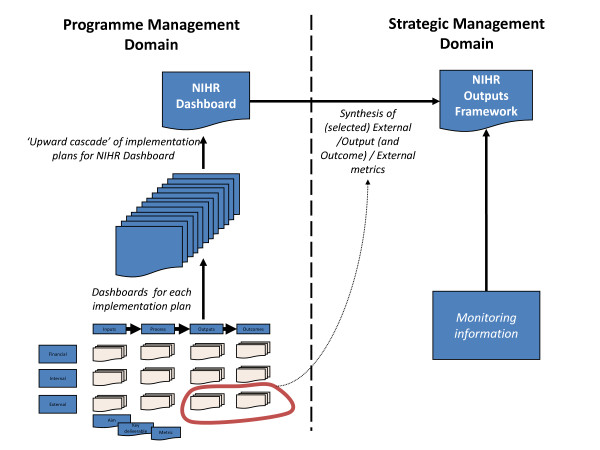
Conceptual approach for developing performance monitoring framework

This dashboard structure was used to create a series of dashboard framework templates or 'dash-cards' (Figure 2) that could be completed by managers for each of NIHR's implementation plans. These templates included aims, deliverables, metrics and reporting frequencies for each of the logic model stages in each of the three Balanced Scorecard domains. Our intention was to cascade the performance indicators from these programme-specific dashboards into a system-wide dashboard of indicators (Figure 3), allowing for comprehensive coverage of all relevant research system activity - whilst also creating bespoke performance reports for each of NIHR's programmes of work.

### Stakeholder validation and piloting

Following its initial development we validated the conceptual framework through a series of semi-structured interviews and focus groups with key senior stakeholders within NIHR. We used a "suitability, feasibility, acceptability" approach to assess stakeholders' perceptions of the framework's suitability (fitness for purpose), feasibility (how likely it was that it could be implemented) and acceptability to important stakeholder groups (including researchers, research administrators and research users). We began by presenting the framework in individual interviews with four (out of seven) senior managers from the Department of Health's Research and Development Directorate's Senior Management Team. Following feedback from these managers we presented an updated framework to members of the NIHR Advisory Board, who were invited to comment on its feasibility, suitability and acceptability. We then undertook nine focus groups, conducted in parallel at an away day involving all members of the Research and Development Directorate at the Department of Health. These focus groups allowed us to canvas the opinions of all health research system managers responsible for the implementation of NIHR's operating strategy.

Following the stakeholder feedback outlined above, the framework was piloted across one of NIHR's four main divisions (National Research Programmes). The piloting exercise allowed us to assess the practicality of applying the framework more widely.

### Wider application of conceptual framework across health research system

Managers with direct responsibility for delivering NIHR's implementation plans were subsequently asked to develop performance indicators by completing dash-cards for the plans they were responsible for.

We encouraged indicators to be developed in consultation with the teams responsible for delivering each implementation plan, including contractors and collaborators from outside the Department of Health and NIHR family. This engagement represented the 'bottom-up' aspect of our approach. Its aim was for managers and delivery teams to identify 'ideal' performance indicators for each implementation plan, creating a dashboard of indicators for each work-stream.

Training in completing the dash-cards was provided through the provision of a single workshop to which all implementation leads were invited, supplemented by written instructions to implementation leads. Submitted dash-cards were then reviewed by members of the project team (JG, MH, and AET) and re-iterated with implantation leads where they had requested assistance in applying FABRIC criteria. In addition, face-to-face support was provided to implementation leads where they specifically requested assistance from the project team in applying the conceptual framework to their implementation plans.

### Information architecture of system-wide performance monitoring framework

It was our intention that work-stream specific dashboards would form the basis of a wider NIHR performance monitoring framework, with indicators from these dashboards underpinning the development of two distinct performance reports: the NIHR-wide Dashboard and the NIHR Outputs Framework (as shown in Figure 3).

The system-wide dashboard was designed to act as an internal management tool, providing the Department of Health's Research and Development Directorate with a periodic overview of the performance of NIHR's constituent programmes to guide short term (i.e. quarterly) decision making. As a result, it was envisioned that this report would be populated by programme management indicators derived primarily from work-stream input, process and output indicators, which by their nature relate more readily to early phase system activity.

A separate NIHR Outputs Framework was proposed for external publication to facilitate strategic review and public accountability. It was planned that this report would combine selected output and outcome performance metrics (strategic management indicators) with monitoring information and other modalities of evaluation (e.g. structured case study reports and commissioned external reviews of implementation and impact) to provide a more strategic perspective on NIHR's progress in achieving its over-arching goals. Our work sought to contribute to the development of this strategic performance report by providing metrics and a broader performance information architecture. The work to develop this report was, however, formally incorporated into NIHR's evaluation framework and thus is not discussed here.

### Development of a system-wide performance dashboard

Following completion of the work-stream dashboards we compiled a long-list of all proposed metrics and the information required to construct these metrics (metadata), including their reporting frequency. We then applied a process of aggregation, compounding and selection to create a short-list of thirty metrics as key indicators that might contribute to an NIHR-wide oversight dashboard. This process followed the principles of: aggregating compatible metrics wherever this was meaningful (e.g. all research programmes had proposed measures relating to the percentage of programme funding that had been successfully disbursed within a given time period); compounding commonly proposed metrics to create indicators of efficiency and productivity (e.g. cost per unit measures); and selecting specific indicators for promotion to the NIHR-wide short-list where a measure was considered to be critical to the delivery of one of NIHR's strategic goals (e.g. total number of researchers and trainees supported by NIHR). At this stage we did not exclude indicators that were only reportable at intervals greater than 6 months however frequency of indicator reporting was a key consideration in selecting the final dashboard indicators.

These thirty indicators were then presented to the NIHR senior management team who identified, through a process of discussion-based consensus, what they felt were the most appropriate measures for quarterly review. This engagement represented the 'top-down' aspect of our approach and resulted in fifteen indicators being selected. Given that the aim of the dashboard was to support operational decision making by NIHR, the final short-list of measures was also informed by the need to choose only indicators that could be meaningfully reported on a quarterly basis. To quality assure our selection, we again assessed the shortlisted indicators against the FABRIC criteria to ensure the proposed performance monitoring framework was focused, appropriate, balanced, robust, integrated and cost effective [17]. Mechanisms were subsequently proposed for the collation and reporting to the NIHR senior management team of these high-level indicators (organised by strategic goal) over a pilot period of twelve months.

## Results

In mid-2007, when we first developed the NIHR dashboard framework, eighteen implementation plans had been published. Following consultation with NIHR senior managers, it was determined that sixteen of these plans were suitable for our framework. The excluded implementation plans were one-off initiatives that would not produce performance information on an ongoing basis: namely, the high-level descriptive plan for establishing the governance for NIHR and the plan governing transitioning of funding arrangements for NHS research from the pre-NIHR system. Over the lifetime of our work (up to June 2009) NIHR evolved its implementation strategy, resulting in an increase in the number of implementation plans to forty-three, of which thirty were active in the last six months of our work.

The conceptual framework and indicator development templates were well received by NIHR's senior management team, the NIHR advisory board and the Department of Health's Research and Development directorate. All groups endorsed the approach for piloting. The pilot of the implementation of the framework demonstrated a high degree of user acceptability, with all operational managers able to complete the templates and iterate proposed indicators in line with the FABRIC principles. The piloting process demonstrated that the conceptual framework could be readily applied by managers in NIHR's health research system. It also provided practical experience on applying the framework that could be passed on to other NIHR managers involved in the next stage of implementation.

Completed indicator development templates were received for twenty-four out of thirty work-stream managers within six months of distribution. The twenty-four templates identified exactly two-hundred and fifty different metrics (Table 1).

**Table 1:** Number of proposed long-list metrics by logic-model stage and modified balanced scorecard domain

	Input	Process	Output	Outcome	Total
**Financial**	20	17	17	16	**70**
**Internal**	29	25	23	10	**87**
**External**	17	23	36	17	**93**
**Total**	**66**	**65**	**76**	**43**	**250**

**Figure 4:**
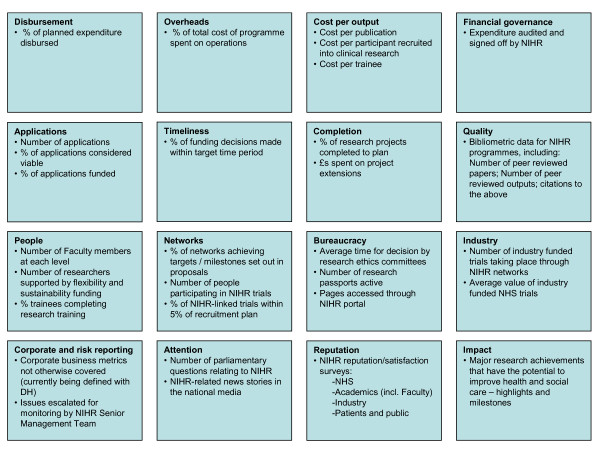
Short-list of performance indicators considered for NIHR-dashboard

Following our process of compounding, aggregation and selection, this long-list of 250 potential metrics was reduced to a shortlist of thirty potential metrics grouped into sixteen categories (Figure 4). Following involvement of the NIHR senior management team, fifteen indicators were selected for initial piloting in an NIHR-wide oversight dashboard (Table 2, Table 3). Consensus was readily achieved by the NIHR senior management team around which indicators should be piloted, aided significantly by reassurances that senior managers would be able to re-iterate the constitution of the dashboard over a period of not less than nine months.

**Table 2:** Number of indicators initially piloted for NIHR-wide oversight dashboard (June 2009) by logic-model stage and modified balanced scorecard domain

	Input	Process	Output	Outcome	Total
**Financial**	-	1	2	-	**3**
**Internal**	1	3	1	-	**5**
**External**	1	1	4	1	**7**
**Total**	**2**	**5**	**7**	**1**	**15**

**Table 3:** Indicators initially piloted for NIHR-wide oversight dashboard (June 2009)

Strategic goal	Indicators	BSC domain	Logic model stage
***Goal 1: Establish the NHS as an internationally recognised centre of research excellence***	Number of applications to NIHR Faculty and Research programmes	External	Input
	
	Number of industry funded trials taking place through NIHR networks and experimental medicine research facilities	External	Process
	
	Total value of industry funded trials taking place in the NHS	External	Output
	
	% of NIHR-portfolio trials achieving greater than 95% of recruitment plan	Internal	Process
	
	Number and favourability of news stories relating to NIHR and NHS research in national and international media each month	External	Output

***Goal 2: Attract, develop and retain the best research professionals to conduct people-based research***	Number of faculty members at each level of NIHR Faculty (Investigators, Trainees and Associates)	Internal	Process
	
	% of personal award scheme (research fellowship) applicants coming from university departments rated as excellent in the 2008 UK Research Assessment Exercise	External	Outcome

***Goal 3: Commission research focused on improving health and care***	% of planned research expenditure disbursed	Financial	Output
	
	% of applications for research grants deemed fundable that were funded	Financial	Process
	
	Number of new publications that attribute NIHR funding deposited on UK PubMed Central each month and % of these in journals targeted at practitioners	External	Output

***Goal 4: Strengthen and streamline systems for research management and governance***	Average time from commission to commencement of NIHR-portfolio studies	Internal	Process
	
	Number of research passports active	Internal	Output
	
	Pages accessed through the NIHR portal and website	External	Output

**Goal 5: Act as sound custodians of public money for public good**	% of total cost of programme funding spent on research administration	Financial	Process
	
	Number of risk register issues escalated for monitoring by NIHR Senior Management Team	Internal	Input

In addition to the production of a suite of indicators suitable for adoption in a system-wide dashboard, the process of developing and implementing our performance monitoring framework contributed directly to the development of a NIHR performance information system and led to the establishment of an executive position within NIHR of a Head of Business Intelligence.

## Discussion

### Rationale for developing a novel framework

In developing our framework our aim was to produce a system for defining performance indicators that would allow managers to monitor the performance of their health research system in an effective and timely manner. We wished to ensure that the set of these indicators was broad enough to provide a balanced perspective of performance, whilst being focused enough that managers felt able to draw insights of use in day-to-day management without suffering information overload.

There is a growing body of work proposing approaches to the evaluation of health research systems [12,14,21-25]. However, at the time of our work, very few of these approaches had been applied empirically. Furthermore, none of the approaches we encountered addressed the needs of health research system managers and policy-makers by providing a mechanism for prospective monitoring of the performance of health research systems. Specifically, our literature review retrieved no performance monitoring frameworks that had been applied to health research agencies with a scope of responsibility comparable to NIHR. We thus felt it necessary to develop a novel framework.

### Design of our framework and relationship to previous work

Basing our framework on two well-established and complementary performance monitoring frameworks allowed us to draw key strengths from both. The use of logic models is not controversial, having been widely practiced for over twenty years in many settings, and their use and benefits in shaping systems for evaluating the impacts of health research have been well described [22-24]. Their potential for providing a prospective framework for monitoring performance in health research systems had not, however, been previously explored. By monitoring resources, activities and effects throughout the logic chain we sought to create a balanced picture of performance across the chain of system activities over time. Such a longitudinal perspective is especially important when assessing performance in research activity, where the lag time between investment and outcome can be extensive [25]. A longitudinal perspective is also useful in providing a basis for observing and understanding the relationship between upstream research activity (inputs and processes) and desired external changes (outcomes and impacts such as change in population health status).

Our decision to combine the logic chain with a modified balanced scorecard was born out of a desire to ensure that our framework encouraged a balanced view of research system activity 'cross-sectionally' as well as longitudinally. By this we mean that we wanted to make sure that indicators reflecting the variety of domains of research system activity, (such as financing, research governance and managing external relationships), were also included.

### The influence of framework design and management preference in indicator selection

The final set of performance measures selected for piloting in the NIHR oversight dashboard demonstrated the effects of two distinct developmental influences:

1) the effect of applying our conceptual framework to the operational plans of a health research system; and

2) the effect of existing performance reporting mechanisms and managerial preferences on the selection, balance and overall character of performance information.

That both the long-list of potential metrics and the final pilot indicators span the full range of logic-model stages and balanced scorecard domains demonstrates the breadth and balance that applying our hybridised framework can achieve. On the other hand, the particular mix of indicators selected to populate the NIHR oversight dashboard demonstrates the focus of senior managers on external and early phase effects of research system activity. In general, the indicators are early phase (input, process and output), in response to the prospective reporting needs of managers and the desire for close monitoring of performance (in this case quarterly). The relative lack of financial indicators in the pilot oversight dashboard relates primarily to the existence of alternative financial reporting mechanisms within the Department of Health for monitoring and accounting for expenditure through NIHR.

### Engagement of health research system managers in design and implementation of performance monitoring frameworks

In many government systems, health research system managers are increasingly being asked to take significant responsibility for the efficiency and effectiveness of national research efforts. Yet often they find themselves managing these systems with a paucity of appropriate performance information. As the end-users of such performance information, it is important that they are involved in the selection and design of reporting mechanisms for such information. Our review of the publicly accessible literature on performance monitoring and evaluation of health research systems suggests that such involvement is not a hallmark of work in this field to date.

Although our engagement with managers and policy-makers in the English Department of Health and NIHR might have been more extensive - for example by undertaking additional cycles of iteration of proposed indicators with implementation plan managers - we still feel that it played a critical role in shaping the outcome of our work, both in terms of the indicators proposed and selected, and in terms of the acceptance of these performance indicators.

### Impact of stakeholder mix on development and selection of performance indicators

In developing and piloting our framework, we encouraged the involvement of stakeholders from the wider health research system by inviting implementation plan leads to involve other constituents as they felt appropriate. Three factors influenced our decision to employ this 'cascade' approach to involvement. First, we felt that such involvement might help broaden consensus on the development of specific indicators with those individuals who would ultimately be performance managed in light of these indicators; (primarily, health research system managers). Second there was a pragmatic need to engage across a large organisation and stakeholder pool quickly with limited resources. Finally, we felt that health research system managers responsible for delivering individual implementation plans were better placed than us to identify which stakeholder groups could best comment on the development of programme specific metrics.

We hoped that stakeholders such as researchers, health system managers, clinicians and patient groups would become involved where appropriate, however we intentionally did not seek to mandate directly the specific involvement of any particular group. The net effect of our cascade approach was that the voices of researchers, clinicians, health system managers and citizens were only present in discussions around the development of particular metrics when such stakeholders were already embedded organisationally within NIHR structures.

Whilst the mix of stakeholders involved was skewed towards health research system managers, we do not believe that this mix had a substantive effect on the type of indicators ultimately selected for the NIHR-wide quarterly performance dashboard in terms of their logic model stage. As noted previously, the mix of indicators piloted in the NIHR-wide dashboard consisted mainly of process and output indicators (such as the number of individuals supported by NIHR Faculty funding or the number of industry funded trials supported by NIHR infrastructure funding). This balance of indicators probably reflected the need to create a prospective performance report that would guide short-to-medium term (quarter-to-quarter) decision making. Given this context, it was always expected that programme management indicators would predominate this report. It was intended that strategic indicators of performance, such as changes in health service practice or improvements to population health, would be captured by the NIHR Outputs Framework (Figure 3).

Although we hypothesise that stakeholder mix did not significantly influence logic model stage prevalence in the final system-wide dashboard, it may be that external stakeholders would have placed greater emphasis on selecting indicators belonging to the external balanced scorecard domain. It is worth noting, however, that of the fifteen indicators proposed for piloting in the NIHR-wide dashboard, nearly half (seven) were from the external BSC domain, compared with three financial and five internal.

Another important question is whether involving a different mix of stakeholders in developing indicators would have produced a substantively different result in terms of the specific detail of proposed indicators. It could be hypothesised that greater involvement of research users (healthcare managers and clinicians for example) or intended beneficiaries of research (e.g. patients, tax-payers) might have led to a greater focus on indicators relevant to health system outcomes.

Although we cannot be sure, we strongly suspect that changing the stakeholder mix at the point of indicator development would *not *have had a direct impact on the range and nature of proposed indicators. We assert this because the strategic focus for indicator development (the goals and priorities against which performance would need to be measured), had already been established in a national health research strategy [16] and encapsulated operationally in NIHR's implementation plans (Appendix 1). Any individual applying our methodology would have been obliged to base indicator development on the objectives and plans set out in the implementation plans, limiting their capacity to substantially redefine the focus of performance assessment.

It is conceivable that broader involvement of stakeholders beyond health research managers might have indirectly affected indicator development, if such engagement led to NIHR altering its operational intent and thus amending its implementation plans. For this to have taken place, however, we postulate that significant dissonance would have been needed between the priorities of stakeholders and the strategic priorities set out in the national health research strategy for England [16]. We feel that it is unlikely that such extensive dissonance existed the time of our work, given that the English health research strategy had been informed by a broad consultative process [26] - and, as noted by Hanney and colleagues, "the overall direction of the proposed new strategy received considerable support during the consultation" [27].

Whilst we feel that the involvement of different stakeholders might not have substantively changed the nature of indicators ultimately selected for the system-wide performance dashboard, we are aware that it may have affected the perceived validity of the structure and outputs of our framework. It may be that in time, a perceived lack of engagement of certain stakeholder groups (such as researchers or clinicians) may undermine the credibility of the approach to performance monitoring by these and other groups. Factors mitigating against this are the involvement of these groups in the development of the antecedent health research strategy, and the previously mentioned existence of individuals from these stakeholder groups within the operational infrastructure of NIHR.

In other health research systems the mix of stakeholders involved in the indicator development process may well have a more significant effect on the final output of such a process than we have postulated was the case with NIHR. In contexts where strategic planning of a health research system has been less consultative or has not explicitly occurred (e.g. where a system has developed incrementally through the evolution of pre-existing components into a single system under common oversight without a specific restatement of strategic intent such as a national strategy on research for health), broadening the mix of stakeholders involved in the indicator development process may well have a significant and beneficial effect on both the development of performance indicators as well as the development of consensus around system function.

### Impact on organisational decision making

The scope of this phase of our work was to develop and apply a conceptual framework for defining appropriate key performance indicators for NIHR. As such, it did not extend to monitoring the impact of the dashboard on organisational decision making beyond this development phase. Nor did it extend to monitoring the iteration of the dashboard and performance reporting system that we expected to occur over time. We hope that in time the experiences of NIHR in applying and refining the dashboard as a system-wide performance assessment tool will be made available to inform the work of others. Our work did, however, have tangible effects on the organisational development of NIHR. These included informing the specifications of NIHR's performance information systems, and the creation of a formal position within NIHR for a Head of Business Intelligence, with responsibility for managing NIHR's performance monitoring and evaluation function.

### Proportionality and focus of our approach

As highlighted in Appendix 2, an important criterion for our work was that it had to balance the benefits of collating performance information against the costs (transaction and opportunity costs) of reporting. It was our initial intention to develop a prospective performance report (i.e. system-wide dashboard) that could provide a balanced 'at-a-glance' perspective of health research system performance, whilst also limiting reporting burden to the minimum necessary level to support such a report. To this end, we can claim some success, in so much as our final proposed dashboard consisted of only fifteen indicators to oversee the activity of a health research system with an annual budget in 2007 of £859 m (US$1.7bn in 2007) [28]. Furthermore, our dashcard-based development process instilled a consistent information architecture in the definition of performance indicators across all of NIHR's activities, the net effect of which was to allow the rapid formulation of performance dashboards at any organisational level that might be required.

In order to fully demonstrate that our performance information architecture was truly proportionate, however, we need to not only demonstrate that the associated reporting burden was low and reporting activity appropriately focused, but also that the overall performance monitoring system was still effective. As highlighted above, we are not currently in a position to comment on the success or otherwise of the system-wide dashboard in enhancing system performance, but we are eager for this insight to become available in time.

In terms of demonstrating that our approach resulted in the development of an appropriately focused set of indicators, we can draw some encouragement from the fact that indicators for the system wide dashboard could be readily mapped each of NIHR's five strategic goals (Table 3). We also feel that it is encouraging that these indicators spanned all of the balanced scorecard and logic model domains and received assent from the NIHR senior management team for piloting. Again, though, definitive conclusions regarding the focus and balance of indicators proposed can only be made by studying experiences of using the dashboard over a period of time.

### Implications for institutional learning

As noted above, our framework omits the learning and growth dimension of the balanced scorecard - and there were good reasons for such an omission. Nevertheless, it may be useful to represent learning processes if such a framework is applied more widely. The effectiveness of stewardship of a health research system is likely to be impaired if the overseeing organisation is ineffective at collating and analysing the consequences of its actions. While the organisation may have a clear grasp of the outputs of the health research system, it will be unsure how its actions contributed to these outputs; to use the well-known terminology of Agryis and Schon, 'single loop' learning will be present, but 'double loop' learning will be absent [29].

The creation of a framework in isolation is unlikely to generate such learning - or to trigger effective performance management as such. The organisation in question must also have the ability to comprehend, reframe and act on the information provided by the framework. This ability, or 'absorptive capacity', may be created by a combination of substantive knowledge, organisational culture and directed leadership [30]. For reasons explained above, our work with the NIHR was not accompanied by attempts to develop such absorptive capacity, yet they are likely to contribute significantly to the successful implementation of performance monitoring frameworks.

### Risks associated with our approach and process lessons learned

The risks associated with our approach for developing performance indicators fall into two categories: 1) those generic risks associated with the development of performance indicators for any system (e.g. perverse incentivisation, loss of rich narrative, etc.); and 2) those risks that occur specifically as a result of applying our particular method of indicator development.

A number of generic challenges (i.e. those not peculiar to the implementation of our framework) face any actor attempting to define performance indicators for a health research system. We believe that foremost amongst these is ensuring that there is sufficient agreement on, and articulation of, the strategic intent of the health research system. Without clarity around the aims and objectives of a health research system, it is impossible to meaningfully measure progress towards the achievement of the system's goals. Although we accept that this statement is true of developing performance information for any system, our experiences of working with health research organisations and systems indicates that lack of strategic clarity remains a critical barrier to higher quality performance monitoring systems. Indeed, often the development of performance monitoring frameworks can highlight areas of strategic inconsistency (e.g. statements of strategic intent unsupported by programmes of activity or programmes of activity that do not correlate to any strategic goals). Although this was not our experience of working with NIHR, we believe that the link between strategy and performance monitoring frameworks represents the greatest vulnerability to those seeking to develop effective performance indicators. This risk does, however, pose a particular challenge to anyone wishing to apply our framework in a different context. Given that our entire approach is predicated on the assumption that a coherent and broadly supported health research system strategy already exists before our framework can be applied, it may be difficult (but by no means impossible) for others seeking to replicate our work in less mature health research systems to achieve comparable results. In such a scenario, it would be important for the process of developing a performance management framework to be intimately linked with the process of developing and refining a system strategy.

The second major generic challenge relates to managing the risks associated with specific indicator development, most critically the risk of creating perverse incentives that skew behaviour of those whose performance is being assessed in a manner not predicted or desired by those responsible for defining performance measures. Once indicators themselves become the focus of behaviour they may disrupt rather than enhance performance. To some degree this risk comes about as a result of the inherently abstractive nature of performance indicators. However, the risk can be reduced by ensuring that performance is interpreted in the context of a balanced set of indicators. The use of a framework for quality assuring the performance indicator development process (such as the FABRIC approach utilised in our approach), combined with a commitment to periodic review and iteration of performance indicators with stakeholders, can go some way to reducing the risk of perverse incentives. We would thus strongly recommend that the adoption of such practices be considered an essential component of any programme to develop a performance monitoring framework for health research systems.

The final generic challenge relates to managing the 'social' threat posed to those who might be held accountable by new models of performance information. Performance monitoring is an imperfect art. Any picture of performance painted by a set of performance indicators will always be an abstraction of real performance, and as such will always be subject to the vagaries of incomplete assessment, sampling error and injudicious interpretation. Nonetheless, the intent when producing performance information is to provide insight that will help guide management (i.e. resource allocation including the allocation of incentives and sanctions). The intellectual limitations of a performance monitoring methodology carry real world consequences to the beneficiaries of the system under consideration. In the context of health research systems, the development of inappropriate performance indicators can have profound impacts upon those regulating, commissioning, conducting and interpreting research. Concern that inappropriate measures may be developed or that measures will be used injudiciously can create significant organisational resistance to the development and implementation of new performance indicators.

In addition to the generic challenges facing those seeking to develop performance indicators for health research systems, we believe that there are some specific challenges associated with implementing our particular framework. The greatest challenge we faced in implementation (one we failed to fully appreciate and mitigate) was the need to provide appropriate ongoing dialogue and support between the project team and the health research system managers tasked with developing indicators for each implementation plan.

We had identified lack of 'buy-in' from implementation plan managers as a key risk to the successful application of our framework within NIHR. We had also anticipated that a novel approach to developing performance indicators would require a greater degree of implementation support than might be expected, had we chosen to implement a framework that was more widely recognised (e.g. a classic balanced scorecard approach). As a result, we provided a training workshop, written instruction and additional support, which led to a number of presentations to operational units to guide the development of implementation plan dash-cards. In spite of these efforts, we were not able to meet with all managers and teams that were ultimately asked to developed indicators.

In retrospect, this failure of communication on our part may have played a significant role in limiting the response rates to our dissemination of the indicator development templates following the initial piloting of dash-cards. It is likely that some health research system managers did not feel comfortable with applying the conceptual framework to their implementation plans (a lack of 'skill') and/or were not convinced of the benefits of utilising our hybrid framework and subsequent information architecture (a lack of 'will'), which may have lowered submission rates of performance dash-cards.

Were we to repeat this exercise, we would pay greater attention to the manner in which we advocated our specific approach to developing performance indicators, and we would prioritise project resources to provide a more comprehensive communications and implementation support function.

## Conclusions

Whilst our work represents only a modest development in conceptual frameworks for performance monitoring, its successful pilot adoption and implementation provides some empirical experience on how theories of performance monitoring for health research might translate into practice. Our framework will be of interest to others seeking to develop performance indicators for health research systems, especially at the national and international levels. Although the specifications set for developing our framework were formally based on a framework developed by the UK government, we feel that they are similar enough to the requirements of other national health research funders as to render the framework relevant to such funders.

Applying our methodology to other health research systems is likely to yield very different performance indicators from those proposed for NIHR. For example, for other research systems at different stages of maturity it may be appropriate to incorporate all of Kaplan and Norton's original BSC domains in the base framework. Similarly, different health research system managers may opt to select a differing mix of indicators for high-level monitoring to those selected by NIHR. This capacity for variation in the specific outputs of applying our framework to different systems is, we believe, entirely appropriate and a key strength of the framework. The hybrid framework does not attempt to provide a 'cookie-cutter' approach to selecting performance indicators, but instead acts as an aid that focuses the development of performance indicators around the strategic objectives set by individual health research systems. As with other performance monitoring frameworks, this means that successful implementation is dependent upon the clarity of strategic objectives set out by a health research system; something that cannot always be taken granted - as the history of health research policy in England demonstrates [28].

In many government systems, health research system managers are increasingly being asked to take significant responsibility for the efficiency and effectiveness of national research efforts. Unfortunately, they often find themselves managing these systems with a paucity of appropriate performance information. As the end-users of such performance information, it is important that they are involved in the selection and design of performance indicators and reporting mechanisms.

The strengthening of the practice of performance monitoring of health research systems will be important for improving the impact that such systems have in meeting national health and economic needs. In publishing our methodology and results in this paper, it is our hope that we might stimulate further commentary from academic and practitioner communities and encourage others to come forwards with their own experiences of performance monitoring of health research systems. Greater sharing of and learning from such practical experience is an important stepping stone to creating performance monitoring systems that can help bridge the gap in our understanding of how health research system activities relate to health research system impacts. Undoubtedly, the academic disciplines of health services research, public health, public sector policy and the evaluation and management sciences have much expertise to contribute to this endeavour. Nevertheless, for academic insight to have greatest impact, it will need to be tested practically in partnership with policy-makers and managers responsible for the operation of national health research systems. Empirical testing of this kind will slowly help create a base of experience and evidence to inform ever more useful performance monitoring approaches. This in turn offers the promise of moving performance monitoring and evaluation of health research systems from the domain of advocacy to genuine performance management for public gain.

## Competing interests

AET was seconded to work at the Department of Health during this work between June 2007 and July 2009; his salary costs were met by a Department of Health Policy Research Programme Award.

## Authors' contributions

JG, MH and TL undertook the literature review and initial development of the performance monitoring framework. JG, MH and TL subsequently worked with AET to refine and implement the framework. AET prepared the manuscript and JG, MH and TL provided significant contributions to its revision. All authors read and approved the final manuscript.

## Appendix 1: Best Research For Best Health Implementation Plans - June 2006

The National Institute for Health Research

Implementation Plan 1.1 The National Institute for Health Research

Implementation Plan 2.1 Funding transition

National Institute for Health Research Faculty

Implementation Plan 3.1 National Institute for Health Research Faculty

Research systems and governance

Implementation Plan 4.1 Bureaucracy busting: Governance, advice and ethics systems

Implementation Plan 4.2 Bureaucracy busting: Research information systems

NHS research infrastructure

Implementation Plan 5.1 Clinical Research Network for England

Implementation Plan 5.2 Clinical research facilities for experimental medicine

Implementation Plan 5.3 Technology platforms

Implementation Plan 5.4 NIHR School for Primary Care Research

NIHR projects, programmes, units and centres

Implementation Plan 6.1 Overview of NIHR research projects, programmes, units and centres

Implementation Plan 6.2 Research for Patient Benefit (RfPB) and Research for Innovation, Speculation and Creativity (RISC) project schemes

Implementation Plan 6.3 Existing R&D programmes

Implementation Plan 6.4 Invention for Innovation Programme

Implementation Plan 6.5 Programme grants for applied research

Implementation Plan 6.6 Research units

Implementation Plan 6.7 Research centres

## Appendix 2: Criteria for performance monitoring and evaluation of NIHR

FABRIC properties of good systems of performance information:

▪ ***Focused ***on the organisation's aims and objectives;

▪ ***Appropriate ***to, and useful for, the stakeholders who are likely to use it;

▪ ***Balanced***, giving a picture of what the organisation is doing, covering all significant

▪ areas of work;

▪ ***Robust ***in order to withstand organizational changes or individuals leaving;

▪ ***Integrated ***into the organisation, being part of the business planning and management processes;

▪ ***Cost Effective***, balancing the benefits of the information against the costs

FABRIC properties of good performance measures:

▪ ***Relevant ***to what the organisation is aiming to achieve;

▪ able to ***Avoid perverse incentives ***- not encourage unwanted or wasteful behaviour;

▪ ***Attributable ***- the activity measured must be capable of being influenced by actions which can be attributed to the organisation, and it should be clear where accountability lies;

▪ ***Well-defined ***- with a clear, unambiguous definition so that data will be collected consistently, and the measure is easy to understand and use;

▪ ***Timely***, producing data frequently enough to track progress, and quickly enough for the data to still be useful;

▪ ***Reliable ***- accurate enough for its intended use, and responsive to change;

▪ ***Comparable ***with either past periods or similar programmes elsewhere; and

▪ ***Verifiable***, with clear documentation behind it, so that the processes which produce the measure can be validated.

Commitments related to performance monitoring and evaluation in 'Best Research for Best Health' - the English National Health Research Strategy (2006)

▪ "We will publish research performance data for NHS Trusts relating to patient numbers, speed and quality, to provide a source of information on reliability on which to base judgements about the locations of clinical trials." (p12)

▪ "Monitor the advancement and assess the effects of public involvement in NHS, social care and public health research." (p24)

▪ "Implement methods of evaluating the equality and effectiveness of our capacity, infrastructure and direct funding." (p32)

▪ "All elements of the new strategy will operate under clear and robust management arrangements supported by programmes of evaluation, including ... regular review of outputs, outcomes and value for money." (p34)
